# A Novel Immunoglobulin-Immunoglobulin Interaction in Autoimmunity

**DOI:** 10.1371/journal.pone.0001637

**Published:** 2008-02-20

**Authors:** Shigeyuki Kawa, Kei Kitahara, Hideaki Hamano, Yayoi Ozaki, Norikazu Arakura, Kaname Yoshizawa, Takeji Umemura, Masao Ota, Sadaaki Mizoguchi, Yasunori Shimozuru, Seiamak Bahram

**Affiliations:** 1 Center for Health, Safety and Environmental Management, Shinshu University, Matsumoto, Japan; 2 Department of Medicine, Gastroenterology, Shinshu University School of Medicine, Matsumoto, Japan; 3 Department of Legal Medicine, Shinshu University School of Medicine, Matsumoto, Japan; 4 Eisai Company Ltd., Tsukuba Research Laboratories, Tsukuba-shi, Ibaraki, Japan; 5 Laboratoire Central d'Immunologie, Pôle de Biologie, Hôpitaux Universitaires de Strasbourg, and Centre de Recherche d'Immunologie et d'Hématologie, Strasbourg, France; Centre de Recherche Public-Santé, Luxembourg

## Abstract

Well over six decades since its first description, the Rheumatoid Factor (RF)—autoantibodies recognizing Fc (constant) portion of IgG through their own Fab (antigen binding variable segments)—is believed to have come of age. Autoimmune pancreatitis is a unique form of pancreatitis, biologically characterized by an elevated serum IgG4 concentration. Given the fact that IgG4 myeloma proteins can act as RF, we initially hypothesized that IgG4 in autoimmune pancreatitis might do likewise, hence potentially contributing to disease pathogenesis. Indeed Western blotting clearly showed that IgG4 binds to IgG1 κ, IgG2 κ, IgG3 κ myeloma proteins, as well as to IgG Fc, in line with a typical RF activity. Further experiments however unraveled the unexpected fact that unlike hitherto known RF, IgG4 does not engage IgG Fc through its Fab, but its very own Fc. These data therefore collectively describe a Novel RF (NRF) in autoimmune pancreatitis. In the future, the relevance of NRF, beyond autoimmune pancreatitis, in both diagnosis/prognosis as well as pathophysiology of autoimmune and other systemic diseases where IgG4's role seems paramount, needs to be systematically assessed.

## Introduction

Despite the identification or the generation of numerous natural and/or transgenic/gene deficient animal models of autoimmunity, the translation of the clinical symptomatology and or biological/immunological pathophysiology to man and vice versa has led to few tangible results in the fields of diagnosis/prognosis and/or treatment of human autoimmune diseases [1], [2]. Perhaps the major hurdle in this quest, at least in man, is the extraordinary complexity, if not uniqueness, of each autoimmune disorder leaving a few common denominators helping to understand the pathology as a whole. One such common denominator are autoantibodies, present–albeit in different titers and against different targets–in virtually all human autoimmune disorders [3]. Among these, the Rheumatoid Factor (RF), along with antinuclear antibodies are the most prominent ones, both in terms of frequency and/or possible role in pathogenesis or yet resolvance of autoimmune diseases. RF are anti-IgG antibodies of mainly IgM but also IgG (or other) isotypes [4]. The interaction between the RF and IgG is well documented; the RF Fab (antigen binding site) recognizes IgG Fc (constant) segment most frequently at the level of Cγ2-Cγ3 segments [5].

Autoimmune pancreatitis is an emerging syndromic entity characterized by several cardinal features distinctive from chronic pancreatitis (cf. *infra*)[6], [7]. The latter, mainly caused by alcohol abuse, can result in severe impairment of exocrine and endocrine pancreatic functions. Imaging findings include pancreatic stones and irregular dilatation of the pancreatic duct. Autoimmune pancreatitis is an alternative form of chronic pancreatitis marked by irregular narrowing of the main pancreatic duct and swelling of the pancreatic parenchyma. The disease is associated with various autoimmune features including hypergammaglobulinemia, histological evidence of lymphoplasmacytic inflammation, occasional coexistence of other autoimmune and/or systemic diseases, and a favorable response to glucocorticoid treatment [8], [9]. Typical clinical features include relative preponderance in elderly male, high frequency of obstructive jaundice-which incidentally together with the swelling of the pancreatic parenchyma can lead to erroneous diagnosis as pancreatic cancer; leading to unnecessary surgery [9]–[11]. They also include extra-pancreatic manifestations i.e. sclerosing cholangitis, [12] sialadenitis, [13] retroperitoneal fibrosis, [14] hilar lymphadenopathy, [15] hypothyroidism,[16] tubulointerstitial nephritis [17], hypophysitis [18] and prostatitis [19]. The variety of these extra-pancreatic lesions suggest, the possibility for autoimmune pancreatitis being a unique link, defining a previously unappreciated systemic disease [14], [20].

Biologically, the most salient feature of autoimmune pancreatitis was our previous report of a specific augmentation of serum IgG4 levels [21]. IgG4, quantitatively the least prevalent of all human IgG subclasses, has two unique features: not being able to activate the classical complement pathway and to bind antigen with low affinity [22]. It has been previously reported to be specifically involved in a number of disorders among which the following are relevant both in terms of prevalence as well as public health. They include parasitic infestation [23], various forms of atopy [24], idiosyncratic drug-induced hepatitis [25], besides the well-established role of anti-desmoglein IgG4 as *bona fide* autoantibodies in pemphigus vulgaris and pemphigus foliaceus [26].

Here we initially aimed to confirm the antigenic reactivity of IgG4 as RF. However the findings detailed below led to the unexpected identification of a novel topology of autoantigen-autoantibody interaction, hereafter called Novel RF (NRF), in contrast to the original RF [27]–[29], consequently re-named Classical RF (CRF).

## Methods

### Patients, controls and diagnostic criteria

Serum samples were obtained from 65 autoimmune pancreatitis patients-54 men and 11 women-aged 38–79 years (median 62.4 years) as well as the following “control” populations: 111 suffering from alcoholic or idiopathic chronic pancreatitis, 96 diagnosed with pancreatic cancer, 40 with autoimmune hepatitis, 39 with primary biliary cirrhosis, 20 with primary sclerosing cholangitis, 13 with systemic lupus erythematosus, 7 with Sjögren's syndrome, 3 with progressive systemic sclerosis and from 130 healthy subjects. All sera were stored at −20°C prior to analysis. All patients with autoimmune pancreatitis fulfilled the revised diagnostic criteria proposed by Japan's Pancreas Society. [30] including the following biological and radiological findings: elevated serum immunoglobulin (including IgG4 as established by single radial immunodiffusion; see below) and/or positive autoantibodies e.g. anti-nuclear antibody and RF (N-Assay TIA RF Nittobo (Nitto Boseki Co., Ltd, Koriyama, Japan) and irregular narrowing of the main pancreatic duct as evidenced by endoscopic retrograde cholangio-pancreatography as well as an enlarged pancreas as assessed by ultrasonography, computed tomography, or magnetic resonance imaging. Histological confirmation of lymphoplasmacytic infiltration and fibrosis in the pancreas was obtained for 13 of these autoimmune pancreatitis patients. All 111 patients with alcoholic or idiopathic chronic pancreatitis had either marked irregular dilation of the main pancreatic duct or calcification of the pancreas. The diagnosis of pancreatic cancer was confirmed by histology in 38 patients and by both typical imaging findings and the clinical course in the remaining 58 patients. All subjects (patients and controls) provided written informed consent for procedures performed in this study which was approved by Ethics Committee of the Shinshu University School of Medicine.

### Western blotting

Throughout experiments, immunoglobulin samples were resolved on 10 % sodium dodecyl sulfate (SDS)- polyacrylamide gels (PAGE) under reducing conditions followed by transfer onto polyvinylidene difluoride membranes (Bio Rad 163-0181, Hercules, CA). The blots were incubated with a variety of monoclonal antibodies (1∶1000 dilutions) (see *infra*) and developed using enhanced chemiluminescence (ECL, Amersham Pharmacia Biotech).

### Antibodies

The following human myeloma immunoglobulins were used: IgG1κ, IgG2κ, IgG3κ, IgG4κ, IgA1κ, IgA2κ, IgMκ (myeloma protein, Binding-Site, Birmingham, UK), IgDκ (myeloma plasma, Calbiochem, La Jolla, CA), IgEλ (myeloma plasma, Athens Research & Technology, Athens GA), IgG F(ab')_2_ (MP Biomedicals, Inc. Aurora, Ohio) and IgG Fc (Athens Research & Technology, Athens, GA) (Fc fragments were produced by papain digestion and affinity chromatography of normal human IgG, itself isolated upon fractionation and DEAE chromatography). The specificity of each immunoglobulin sample was confirmed by Western blotting, where membranes were incubated with the corresponding peroxidase-conjugated antibody for each immunoglobulin isotype. For the totality of experiments the following peroxidase-conjugated second antibodies were used: mouse anti-human IgG1 (Zymed 05-3320, South San Francisco, CA), mouse anti- human IgG2 (South Biotech 9060-05, Birmingham, AL), mouse anti-human IgG3 (South Biotech 9210-05), mouse anti-human IgG4 (South Biotech 9200-05) (of IgG1 isotype which reacts to the Fc portion of IgG4), goat anti-human IgA (Zymed 62-7420), goat anti-human IgM (South Biotech 2020-05), goat anti-human IgD (Biosource AHI0204, Camarillo, CA) and goat anti-human IgE (Biosource AHI0504). Finally, goat anti-human κ light chain (Bethyl Laboratories, Inc, A80115P) was used to detect IgG4 F(ab')_2_.

### Immunoglobulin preparations

In order to use an identical patient serum in all experiments, the autoimmune pancreatitis sera used was indeed a pool derived from all 65 patients (500 µl of each serum×65 = 32.5 ml of pooled serum).The IgG4 concentration of this pooled serum was 755 mg/dl as defined by single radial immunodiffusion (Human IgG Subclass Single Dilution; BINDARID™ Kits; the Binding Site Limited, Birmingham, UK). This value was further used to construct a standard curve for ELISA (see *infra*). To confirm the reactivity of the patients' IgG4 to Fc fractions of IgG1, IgG2 and IgG3; a preparation of IgG Fc lacking IgG4 Fc was needed. For such IgG Fc was first absorbed by affinity chromatography where the column was bound with anti-IgG4 antibody (The Binding Site Limited, Birmingham, UK). The resultant IgG Fc depleted of IgG4 Fc was then used in Western blotting in addition to intact IgG Fc. IgG4 Fc and IgG4 F(ab')_2_ were generated through papain [31] and pepsin treatments [32] of purified IgG4 (extracted from pooled patient sera by affinity chromatography as described above) respectively. The step-by-step methodology is provided in Figure S1.

### ELISA

The capacity of IgG4 to act as RF was assessed by examining its binding capacity for human IgG1 or IgG2 or IgG3 in ELISA. Micro ELISA plates (Nunc immunoplate 446612, Rochester, NY) were coated with human myeloma IgG1κ, IgG2κ or IgG3κ (1 µg/well) proteins. Plates were washed and then blocked with 1% bovine serum albumin (BSA) in phosphate-buffered saline (PBS) containing 10 mmole of ethylene diamine tetra-acetic acid (EDTA). After another wash, serum samples were diluted to 1∶5000 and added. Immobilized complexes were then incubated with peroxidase-conjugated anti-human IgG4 monoclonal antibody in enzyme conjugate stabilizer solution (Stab-ELISA-rHRP Diluent/Stabilizer, Cygnus Technologies I-035, Southport, NC) (1∶2000 dilution). The enzyme bound to the wells was incubated in the dark with tetramethylbenzidine substrate solution (TMB One-component Microwell Peroxidase Substrate, Kirkegaard & Perry Laboratories 53-00-01, Gaithersburg, ML). The reaction was halted by the addition of stop solution (TMB One-Component Stop Solution Kirkegaard & Perry Laboratories 50-85-05) and the plate was read at 450 nm. To construct the standard curve, the pooled sera of autoimmune pancreatitis patients were serially diluted from 39 to 10,000 ng/ml of IgG4 and the optical density for each IgG4 value was plotted. The optical density of bound IgG4 concentration in each serum sample was converted to an absolute IgG4 value using this standard curve. The concentrations of standards were expressed as IgG4 values from the pooled patient sera. The linear correlation was obtained for each assay system between the absorbance of IgG4 bound to each IgG subclass and the IgG4 value from the pooled sera. The intra-assay variation was less than 5.0%, and the inter-assay variation less than 7.5%. The HRP-conjugated second antibody reacted minimally to coated myeloma proteins.

## Results

The additive “autoimmune” in “autoimmune pancreatitis” stems from several lines of (indirect) evidence. These include a significant association of susceptibility to the disease with premier immune loci i.e. the HLA DRB1*0405-DQB1*0401 haplotype [33], [34] and the Fc receptor-like gene 3 (FCRL3) [35] as well as an augmented serum IgG4 concentrations-which correlates with disease activity [21] and parallels an abundant IgG4-bearing plasma cell infiltration of affected tissues [14]. Given the paramount nature of IgG4 elevation in the disease, our effort converged on its relevance and based on the previous knowledge of myeloma IgG4 being able to act as RF, our investigations first aimed to replicate this aspect with this time non-clonal IgG4 present in autoimmune pancreatitis and eventually in other pathological or physiological conditions.

**Figure 1:**
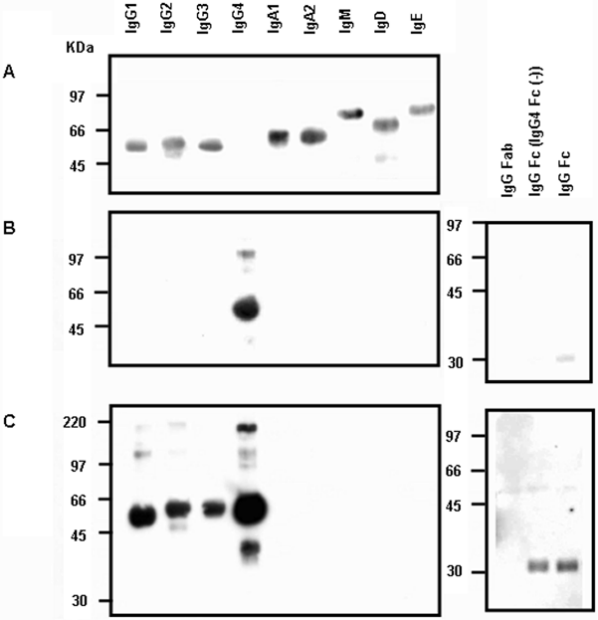
Reactivity of human IgG4 to various immunoglobulin classes and subclasses. Western blot analysis indicates the reactivity of IgG4 in pooled sera of patients with autoimmune pancreatitis to various human immunoglobulin subclasses. (A) The identity of each commercially-purchased immunoglobulin myeloma protein was confirmed upon reaction with the corresponding HRP-labeled anti-isotype antibodies respectively i.e. IgG1κ, IgG2κ, IgG3κ, IgA1κ, IgA2κ, IgMκ, IgDκ and IgEλ (B) Similarly HRP-labeled antibody against IgG4 Fc specifically reacted to IgG4κ. It also reacted to IgG Fc (faint band on the right panel) but not to IgG Fab or yet Ig Fc lacking IgG4 Fc. (C) An identical membrane (to panel A) was subjected this time, first, to pooled autoimmune pancreatitis sera and then to HRP-labeled anti-IgG4 Fc antibody which in contrast to panel B, reacted to IgG1κ, IgG2κ, IgG3κ, IgG Fc and affinity-purified IgG Fc lacking IgG4 Fc, but not to other human immunoglobulins and IgG Fab, hence establishing the fact that patient IgG4 reacts to IgG1,2 and 3 Fc (additional bands at 220 kDa and about 38 kDa are likely aggregation or degradation products of IgG4). Experiments were repeated three times with identical results and the pooled serum was used at 1∶1000 dilution.

We first used Western blotting to explore the Ig–Ig interaction patterns of autoimmune pancreatitis patients' IgG4. We initially were able to show that IgG4 was able to bind to human IgG1, IgG2, IgG3 and IgG Fc but not to human IgA, IgM, IgD, IgE and IgG Fab, hence defining it as a *de facto* RF. In order to do so, we first confirmed the identity of each immunoglobulin myeloma protein used in experiments-IgG1κ, IgG2κ, IgG3κ, IgA1κ, IgA2κ, IgMκ, IgDκ and IgEλ-through reactivity with the corresponding HRP-labeled anti-Ig antibody (Figure 1a). As with other HRP-labeled anti-immunoglobulin antibodies, HRP-labeled anti-IgG4 Fc reacted to IgG4κ myeloma protein and to IgG Fc, but not to IgG Fc lacking IgG4 Fc (Figure 1b). After an identical membrane (as to Figure 1a) was exposed this time first to the pooled sera of autoimmune pancreatitis patients, HRP-labeled anti-IgG4 antibody reacted to IgG1κ, IgG2κ, IgG3κ, IgG Fc, and to a purified IgG Fc lacking IgG4 Fc, but not to other human immunoglobulins or to an IgG F(ab')_2_ (it should be also noted that the reactivity of HRP-labeled anti-IgG4 antibody to IgG4κ seems to be amplified upon exposure to autoimmune pancreatitis sera). These results hence indicate that IgG4 from autoimmune pancreatitis patients is able to bind IgG1κ, IgG2κ and IgG3κ as well as IgG Fc (and perhaps to IgG4κ myeloma protein itself) (Figure 1c) (it should be noted that several individual sera were equally tested and gave similar results as the pooled serum).

**Figure 2:**
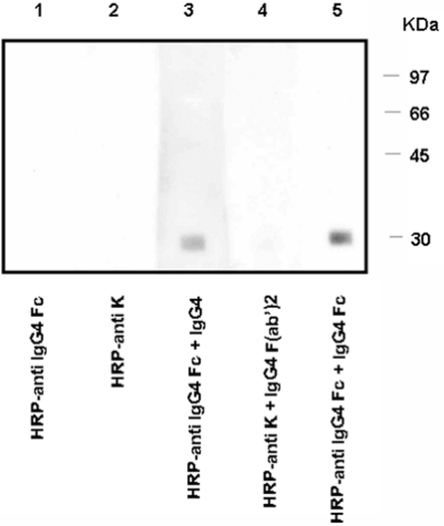
The topology of IgG4–IgG interaction. Western blotting was used to establish whether the Fc or the Fab portion(s) of IgG4 reacted to IgG Fc. IgG Fc lacking IgG4 Fc was blotted on each lane. HRP-labeled anti-human IgG4 Fc or HRP-labeled anti-human κ light-chain showed no reactivity to IgG Fc lacking IgG4 Fc (lanes 1 and 2). HRP-labeled anti-IgG4 Fc antibody reacted to lanes 3 and 5, which were previously incubated with purified IgG4 and IgG4 Fc, respectively. HRP-labeled anti-human κ light-chain had no reactivity to lane 4, which was incubated with IgG4 F(ab')_2_ (lane 4). These results indicated that IgG4 binds to IgG Fc by its own Fc, and not Fab as a classical RF. Experiments were repeated three times with identical results.

Having established that IgG4 recognizes IgG Fc, we next examined whether IgG4's Fc or Fab portion reacts to IgG Fc. A preparation of IgG Fc that lacked IgG4 Fc was resolved on 10 % SDS-PAGE and blotted. As expected, HRP-labeled anti-IgG4 Fc or HRP-labeled anti-κ light-chain showed no reactivity to this preparation (Figure 2, lanes 1 and 2 respectively). However after prior exposure to purified IgG4 or IgG4 Fc, HRP-labeled anti-IgG4 Fc reacted strongly with this IgG Fc lacking IgG4 Fc (Figure 2) whereas HRP-labeled anti-human κ light-chain did not interact with IgG Fc lacking IgG4 Fc after incubation with IgG4 F(ab')_2_ (Figure 2, lane 4). These results collectively indicate that the Fc portion of IgG4, rather than the Fab portion, binds to IgG Fc, and that the binding of IgG4 to IgG is not attributable to the autoantibody activity of IgG4 i.e. via Fab.

**Figure 3:**
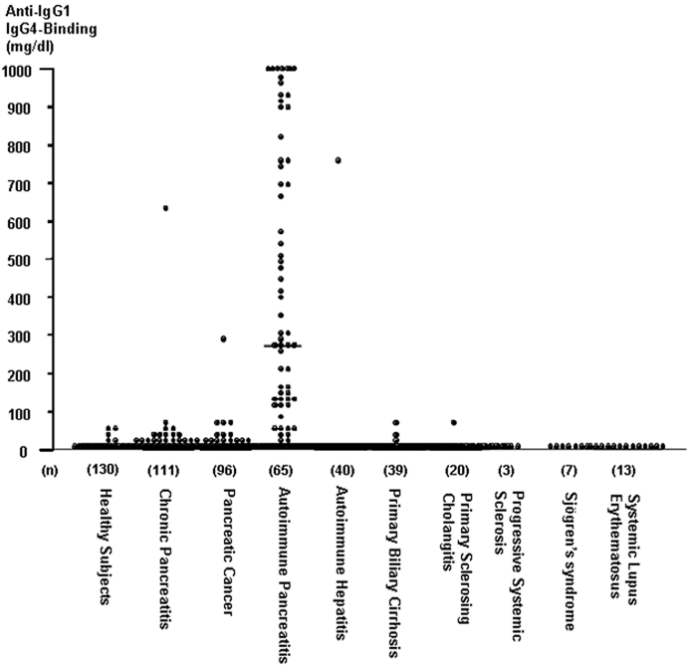
Scattergram of serum levels of IgG4 bound to IgG1κ myeloma protein. Similar results were seen with assay systems for IgG4 bound to IgG2 κ and IgG3 κ myeloma proteins.

**Figure 4:**
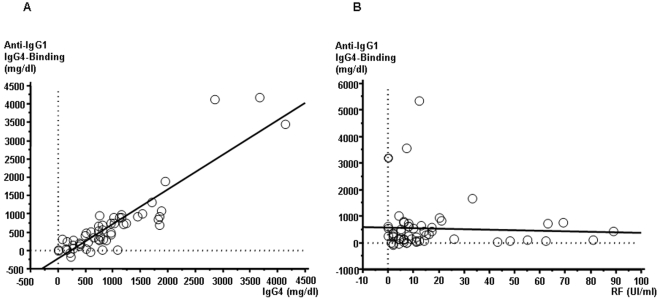
CRF vs. NRF. (a) Correlation between serum levels of IgG4 bound to IgG1 and IgG4 value. A significant correlation was found. (b) Correlation between serum levels of IgG4 bound to IgG1 and those of RF. No significant correlation was found.

This Fc-Fc interaction is a novel finding and therefore we have decided to name it Novel RF (NRF) in contrast to the “original” RF hereafter termed Classical RF (CRF). It was now worthwhile to investigate the relevance of NRF *in vivo*. We first sought to confirm by ELISA that each autoimmune pancreatitis patient's serum IgG4 does act as a Novel RF, by examining its binding capacity to human IgG1 or IgG2 or IgG3. Serum IgG4 bound to IgG1 was undetectable (or at very low levels) in 130 healthy controls as well as patients with other autoimmune diseases (n = 119; cf. supra) including autoimmune hepatitis, primary biliary cirrhosis, primary sclerosing cholangitis, systemic lupus erythematosus and Sjögren's syndrome (Figure 3, Table 1) or other pancreatic affections i.e. chronic pancreatitis and pancreatic cancer; all in contrast to a cohort of autoimmune pancreatitis patients which harbored a significantly elevated concentration of IgG4. Figure 4 clearly depicts two important facts with regards to IgG4: firstly that indeed it does show NRF activity in the sense that its serum concentration is in near perfect correlation with its capacity to bind IgG1 (Figure 4a) and that this NRF activity is in total discordance with that of the CRF i.e. no correlation with IgG1 binding and CRF (Figure 4b). Given the almost perfect correlation of IgG4 concentration to that of the functional IgG1 binding we chose to pursue with this latter as it depicts a functional activity rather than just a “passive” concentration (Figure 4). As depicted in Figure 3 and Table 1 high serum IgG4 concentrations able to bind to IgG1 were detected in most samples from the patients with autoimmune pancreatitis (median 269, range 0–4,185 mg/dl), and the concentrations were significantly higher than those observed in other selected diseases (Figure 3, Table 1). Finally, assays of IgG4 bound to IgG2 or IgG3 showed results that were similar to the results of the assays of IgG4 bound to IgG1 (Table 1). Inversely however neither IgG1, 2 or 3 did show any NRF activity (unpublished data) i.e. IgG subclasses other than IgG4 did not have the ability to bind to IgG1–4.

**Table 1:** Serum levels of IgG4 binding to each IgG subclass in patients with autoimmune pancreatitis and various other conditions.

Patients and controls	Anti-IgG1 (IgG4 mg/dl)		Anti-IgG2 (IgG4 mg/dl)		Anti-IgG3 (IgG4 mg/dl)	
	median (range)	p (v.s. autoimmune pancreatitis)	Median (range)	p (v.s. autoimmune pancreatitis)	Median (range)	p (v.s. autoimmune pancreatitis)
**Autoimmune pancreatitis (n = 65)**	296 (0–4,185)		306 (0–3,968)		142 (0–4,995)	
**Healthy subjects (n = 130)**	0 (0–57)	<0.0001	0 (0–59)	<0.0001	0 (0–0)	<0.0001
**Chronic pancreatitis (n = 111)**	4 (0–635)	<0.0001	0 (0–435)	<0.0001	0 (0–901)	<0.0001
**Pancreatic cancer (n = 96)**	2 (0–289)	<0.0001	2 (0–134)	<0.0001	0 (0–346)	<0.0001
**Autoimmune hepatitis (n = 40)**	0 (0–761)	<0.0001	1 (0–414)	<0.0001	1 (0–214)	<0.0001
**Primary biliary cirrhosis (n = 39)**	1 (0–71)	<0.0001	1 (0–147)	<0.0001	0 (0–24)	<0.0001
**Primary sclerosing cholangitis (n = 20)**	1 (0–73)	<0.0001	5.5 (0–84)	<0.0001	0 (0–97)	<0.0001
**Progressive systemic sclerosis (n = 3)**	0 (0–1)	0.0048	0 (0–0)	0.0041	0 (0–97)	0.0504
**Sjögren's syndrome (n = 7)**	0 (0–6)	<0.0001	8 (0–13)	<0.0001	0 (0–0)	<0.0001
**Systemic lupus erythematosus (n = 13)**	1 (0–2)	<0.0001	1 (0–69)	<0.0001	0 (0–178)	<0.0001

Mann-Whitney test, autoimmune pancreatitis; autoimmune pancreatitis.

## Discussion

Although some preliminary reports had suggested the presence of pathogenic autoantibodies against specific pancreatic antigens, [36], [37] there is little evidence to conclude for IgG4 autoantibodies to have a direct role in the pathogenesis of autoimmune pancreatitis. This is in contrast to the case of pemphigus where recognition of skin autoantigens (desmogleins) by IgG4 is at the origin of the disease process [38]. Accordingly, we explored alternative ways by which IgG4 could intervene in this process. Based on the fact that several monoclonal (myeloma) IgG4 proteins do show the capacity to act as RF [39] we aimed to ascertain whether serum (polyclonal) IgG4 in autoimmune pancreatitis, does in fact act as RF, that is commonly defined as an autoantibody which recognizes IgG Fc through its Fab [3].

**Figure 5:**
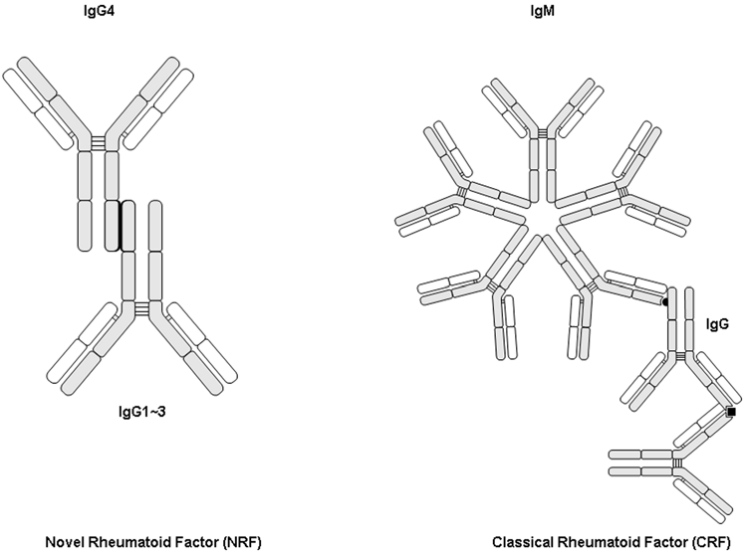
A Novel RF. Schematic representation of two distinct modes of Ig-Ig interaction. On the left: IgG4 Fc interacts with Ig Fc. On the right: IgM RF recognizes IgG in a “classical” Fab-Fc recognition.

Western blot analysis showed that IgG4 did indeed bind to IgG1κ, IgG2κ, IgG3κ and IgG Fc, hence acting, till this stage, as a *de facto* RF. However, and surprisingly, IgG4 binding to IgG Fc was due to its Fc, rather than to its Fab. This is formally inconsistent with the simplest definition of an autoantibody that is an immunoglobulin recognizing a self antigen through its variable, antigen binding Fab, and consequently that of a RF i.e. an autoantibody recognizing IgG Fc. Based on this contention, but unwilling to introduce yet a new nomenclature, we propose for this new Ig-Ig interaction to be called Novel RF (NRF) in contrast to the original finding [27], [28], subsequently called Classical RF (CRF) (it should be noted that originally, the term RF was used as an alternate for “Rheumatoid Arthritis Serum” prior to any knowledge as to its fine identity and structural mode of action; see [29]). Importantly however, any overlap between NRF and CRF could be excluded based on the following arguments: (a) Western blotting shows no IgG4 Fab- Ig Fc (CRF) interaction and (b) ELISA showed that serum level of IgG4 bound to each IgG subclass correlated well with the serum IgG4 level itself (i.e. the totality of IgG4-Ig interaction is through Fc-Fc engagement), and (c) the reciprocal absence of any link between IgG-bound IgG4 levels and RF, excluding therefore any CRF reactivity (Figure 5). It should be also reminded that a previous report, albeit *in vitro*, did show through domain swapping experiments that the binding of a monoclonal IgG4 RF (isolated this time from a rheumatoid arthritis patient) to IgG is through their respective constant segments and not Fab-Fc [40]. This is in direct support of our results obtained on total seric, polyclonal, IgG4. Finally the fact that IgG4 anti-immunoglobulin antibodies have been found in all human beings puts the pathophysiological relevance of the here presented data into perspective [41]. It is equally noteworthy that this IgG4 Fc–Ig Fc interaction extends beyond man to a number of other animal IgG. Some such as mouse, rabbit, guinea pig, bovine and goat, showed strong reactivity with human IgG4. On the other hand, sheep, horse and rat IgG showed scarce reactivity to human IgG4 (unpublished data).

What is the *raison d'être* of NRF? Is it beneficial or detrimental to the disease process? Is it limited to IgG4 or could be found for other IgG subclasses or Ig classes? Is it restricted to autoimmune pancreatitis or of more general relevance ? A few clues could be presented here. Firstly, one should be reminded of some structural specificities of IgG4, paramount among which are its inability to engage C1q, hence preventing it to activate the classical complement pathway and dynamic *in vivo* exchange of Fab arms leading to bispecific antibodies [22], [42] (see *infra*). Secondly, there is presently no reason to support for NRF to be limited to autoimmune pancreatitis and given the relevance of IgG4 in a vast array of other disorders, it is logical to assume NRF involvement therein. These include beside systemic manifestations of autoimmune pancreatitis, the following disorders for most prevalent and relevant both in terms of frequency as well as public health implications. They include membranous nephropathy, a prevalent etiology of end-stage renal failure, is characterized by deposit of IgG4 along the epithelia surface of glomerular basement membrane [43]. Along with IgE, IgG4 has been reported to be elevated in several helminthiasis [23] as well as allergic disorders [24]. Finally in line with pemphigus where anti-desmoglein antibodies have been established as pathogenic, patients developing toxic liver hepatitis or idiosyncratic drug induced hepatitis have been shown to carry anti-cytochrome CYP21E IgG4 autoantibodies [25]. Finally it should be noted that NRF is specific of IgG4 as none of the other IgG subclasses were found to show such activity (unpublished data).

In more general terms and despite the fact that is is likely premature to speculate much on the physiological/pathological effects of IgG4 Fc-IgG Fc complexes *in vivo*, several not exclusive situations could be envisioned based on circumstantial evidences. (1) IgG4 Fc-Fc binding may have a pathological role within the inflammatory process, or even induce inflammation *per se*. An example among may: In a mouse lupus nephritis model, aggregated immunoglobulins react with fibronectin physicochemically; this reaction in turn causes clustering of integrins and internalization of immunoglobulins by glomerular endothelial cells, resulting in fine in glomerular injury [44]. IgG4 Fc-Fc binding may therefore indeed induce aggregation of immunoglobulins. However, to date, there have been few reports indicating IgG4 deposition in the affected pancreatic tissue, therefore not in support of a direct immnopathological role of IgG4 Fc-IgG Fc binding. (2) Alternatively, IgG4 may aid in the clearance of immune complexes by forming larger complexes that are more effectively cleared. In AIP there are high levels of serum immune complexes that can be linked to complement activation. In addition, we previously found that immune complexes detected by monoclonal rheumatoid factor method also showed IgG4 activity [21]. Possibly, IgG1-type immune complexes trigger the complement activation system via the classical pathway, and that IgG4 contributes to the clearance of immune complexes and the termination of the inflammatory process [45]. (3) Another possibility is that IgG4 could block Fc-mediated effector functions of other IgG1 and IgG3 and may dampen the inflammatory response. (4) Finally it might be of interesting to pursue a recent report with regards to IgG4 where authors document the *in vivo* heavy (and light) chain swapping leading for unique biological properties including cross-linking of 2 distinct antigens. This adds to the already mentioned unique properties of IgG4 and may have physiological, pathological as well as therapeutic consequences (given that a number of therapeutic monoclonal antibodies are of IgG4 isotype).

In conclusion, a sequential set of experiments, aimed initially to confirm the mode of interaction of supposedly RF-like autoantibodies to their cognate autoantigens revealed the unexpected fact that IgG4 autoantibodies interact *in vivo* with IgG autoantigen in an unprecedented topology. This Fc-Fc interaction departs from the present definition of RF as a Fab-Fc interaction hence the proposed dichotomic nomenclature of CRF vs. NRF. The fact that IgG4 autoantibodies have been universally found in man [41] and in the diverse set of above mentioned pathologies sets the agenda for further characterization of NRF both in physiology as well as pathology.

## Supporting Information

Figure S1 Detailed protocol for generation of IgG fractions.
(0.06 MB DOC)
