# Supplementary material for: A Novel Immunoglobulin-Immunoglobulin Interaction in Autoimmunity
Source: PLoS One. 2008 Feb 20;3(2):e1637. doi: 10.1371/journal.pone.0001637 (PMC2249926; doi:10.1371/journal.pone.0001637)
Supplement: Figure S1 — Detailed protocol for generation of IgG fractions. (0.06 MB DOC) [file pone.0001637.s001.doc]

**[1] Purification of human IgG**

Starting material: 4.2ml of pooled serum.

↓

Centrifugation (12000 rpm, 15 min.)

↓

Supernatant

↓

Dilute three times with 1M-Glycine/NaOH 0.15M-NaCl pH=8.6

*(Glycine; 077-00735, Wako Pure Chemical Industries, Ltd., JAPAN)*

*(NaCl; 191-01665, Wako Pure Chemical Industries, Ltd., JAPAN)*

↓

Add Protein-A gel into the diluted pooled serum

*(ProSep-vA High Capacity; Millipore Corporation U.S.A)*

↓

Stir overnight at 4°C

↓

Pack into a column, and wash with washing buffer (50mM Tris-HCl 0.15M-NaCl pH=7.5)

*(Tris(hydroxymethyl)aminomethane; 0382Z012-709 GERMANY)*

↓

Wash the column until the protein concentration of the washing buffer is under 280nm=0.01

↓

Dissociate the bound IgG from Protein-A gel with elution buffer (0.1M-Citrate pH=3.0)

*(Citric Acid; 035-03495, Wako Pure Chemical Industries, Ltd., JAPAN)*

↓

Dialyze the eluted solution with 50mM Tris-HCl 0.15M-NaCl pH=7.5 at 4°C

↓

Exchange dialysis buffer four times

↓

Concentrate the dialyzed solution (Centricon Plus-20 (MW=5000); Millipore Corporation, U.S.A)

↓

Concentrated solution is called Protein-A Purified Human IgG

↓

Measure the purity of Protein-A Purified Human IgG (OD280nm, HPLC, SDS-PAGE)

**[II] Preparation of anti-human IgG4 binding CNBr-activated Sepharose 4 Fast Flow**

**CNBr-activated Sepharose 4 Fast Flow**

*(CNBr-activated Sepharose 4 Fast Flow; GE Healthcare Bio-Sciences AB, SWEDEN)*

↓

Hydrate the gel with cold 1mM HCl on the glass filter several times

↓

Wash with 0.1M NaHCO3 0.5M NaCl pH=8.3

*(NaHCO3; 191-01305, Wako Pure Chemical Industries, Ltd., JAPAN)*

↓

Affinity purified sheep anti-human IgG4 (AU009) was dialyzed with buffer and added to the CNBr-activated Sepharose 4 Fast Flow.

*(AU009; The Binding Site Limited, U.K)*

*(Buffer; 0.1M NaHCO3 0.5M NaCl pH=8.3)*

↓

Incubate overnight at 4°C

↓

Add blocking buffer (0.2M Glycine pH=8.0)

*(Wako Pure Chemical Industries, Ltd., JAPAN)*

↓

Incubate for two hours at room temperature

↓

Wash the gel with 0.1M NaHCO3 0.5M NaCl pH=8.3

↓

Wash the gel with 0.1M Acetic Acid 0.5M NaCl pH=4.0

*(Acetic Acid: 017-00256, Wako Pure Chemical Industries, Ltd., JAPAN)*

*(Sodium Acetate: 2111519, KOKUSAN KAGAKU Co, Ltd., JAPAN)*

↓

Wash the gel with 0.1M NaHCO3 0.5M NaCl pH=8.3

↓

Wash the gel with 50mM Tris-HCl 0.15M NaCl pH=7.5

↓

Stock the gel in 50mM Tris-HCl 0.15M NaCl 0.1%NaN3 pH=7.5

**[III] Affinity purification of human IgG4**

Protein-A Purified Human IgG

↓

Add to the anti-human IgG4 binding CNBr-activated Sepharose 4 Fast Flow

*(Affinity Purified Anti-Human IgG4( AU009 ); The Binding Site Limited, U.K)*

*(CNBr-activated Sepharose 4 Flow; GE Healthcare Bio-Sciences AB, SWEDEN)*

↓

Incubate overnight at 4°C

↓

Wash with 50mM Tris-HCl 0.15M NaCl pH=7.5

↓

Elute with 5M NaI

*(NaI: 198-02275, Wako Pure Chemical Industries, Ltd., JAPAN)*

↓

Dialyze overnight with 50mM Tris-HCl 0.15M NaCl pH=7.5 at 4°C

↓

Exchange dialysis buffer four times

↓

Concentrate the dialyzed solution (Centricon Plus-20 (MW=5000); Millipore Corporation, U.S.A)

↓

Concentrated solution is called Affinity Purified Human IgG4

↓

Measure the purity of Affinity Purified Human IgG4 (OD280nm, HPLC, SDS-PAGE)

**[IV] Purification of IgG4 Fc with papain treatment**

Affinity Purified Human IgG4

↓

Dialyze overnight with 0.1M PBS pH=7.2 at 4°C

↓

Add Papain (1/50 of Affinity Purified Human IgG4 concentration)

*(Papain from Papaya Latex (P-3125, 18mg Pro./ml); SIGMA-ALDRICH, U.S.A)*

**↓**

Add L-Cysteine Hydrochloride Monohydrate at final concentration of 10mM

*( L-Cysteine Hydrochloride Monohydrate (033-05272)*; Wako Pure Chemical Industries, Ltd., JAPAN)

↓

Add EDTA-2Na at final concentration of 2mM)

*(EDTA-2Na (345-01865); DOJIN KAGAKU KENKYUSYO, JAPAN)*

↓

Stir occasionally three or four hours at 37°C

↓

Add iodoacetamide at final concentration of 100mM

*(095-02151, Wako Pure Chemical Industries, Ltd., JAPAN)*

↓

React 20 minutes at room temperature

↓

Dialyze three hours with distilled water at 4°C

↓

Dialyze overnight with 0.005M PB pH=7.4 at 4°C

↓

Charge into CM 52 Cellulose Column (0.9 x 50 cm)

*(CM 52; 4037, Whatman Japan K.K, JAPAN)*

↓

Elute with 0.005M PB pH=7.4 (1.2ml/tube/10min)

↓

Measure the protein concentration of eluted solution at OD280nm

↓

Pool the fractions containing protein

↓

Concentrate the pooled solution (Centricon Plus-20 (MW=5000); Millipore Corporation U.S.A)

↓

Dialyze overnight with 50mM Tris-HCl 0.15M NaCl pH=7.5 at 4°C

↓

Charge into ACA-44 Gel (0.9 x 50 cm)

*(ULTROGEL AcA-44; 230161, SEPRACOR, FRANCE)*

↓

Elute with 50mM Tris-HCl 0.15M NaCl pH=7.5 (1.2ml/tube/10min)

↓

Pool the fractions containing protein

↓

Concentrate the pooled solution (Centricon Plus-20 (MW=5000); Millipore Corporation U.S.A)

↓

Concentrated solution is called “Purified IgG4 Fc”

↓

Measure the purity of Purified IgG4 Fc (OD280nm, HPLC, SDS-PAGE), and check IgG4 activity with EIA

**[V] Purification of IgG4 F(ab’)_2_ with pepsin treatment**

Affinity Purified Human IgG4

↓

Dialyze overnight with 0.1M Acetate buffer pH=4.2 at 4°C

↓

Add pepsin (1/50 of Affinity Purified Human IgG4 concentration)

(Pepsin was dissolved in 0.1M Acetate buffer)

*(Pepsin from Porcine Stomach Mucosa (P-7012-250mg, 1/60000); SIGMA-ALDRICH U.S.A)*

↓

Stir occasionally six or seven hours at 37°C

↓

Neutralize with 1N-NaOH

↓

Add 10mM Tris-HCl 0.15M NaCl pH=7.5

↓

Charge into ACA-44 Gel (0.9 x 73 cm)

*(ULTROGEL AcA-44; 230161, SEPRACOR, FRANCE)*

↓

Elute with 50mM Tris-HCl 0.15M NaCl pH=7.5 (1.5ml/tube/5min)

↓

Measure the protein concentration of fractionated solution at OD280nm

↓

Pool the fractions containing protein

↓

Concentrate the pooled solution (Centricon Plus-20 (MW=5000); Millipore Corporation U.S.A)

↓

Add Protein-A (ProSep-vA High Capacity; Millipore Corporation U.S.A)

↓

Stir overnight at 4°C

↓

Pack in the column and elute with 50mM Tris-HCl 0.15M-NaCl pH=7.5

↓

Collect the pass through fractions by washing the column

↓

Concentrate the pass through fractions (Centricon Plus-20 (MW=5000); Millipore Corporation U.S.A)

↓

Concentrated solution is called Purified IgG4 F(ab’)_2_

↓

Measure the purity of Purified IgG4 F(ab’)_2_ (OD280nm, HPLC, SDS-PAGE), and check the activity with EIA.
